# 肺部同时性、持续性多发磨玻璃结节自然病程的全程随访管理策略研究进展

**DOI:** 10.3779/j.issn.1009-3419.2024.106.25

**Published:** 2024-09-20

**Authors:** Chengming HUANG, Yongzhao ZHOU, Yujin FANG, Yanyang LIU, Li WANG, Yu ZHUO, Daxing ZHU

**Affiliations:** ^1^610041 成都，四川大学华西医院全程管理中心; ^1^Integrated Care Management Center, West China Hospital, Sichuan University, Chengdu 610041, China; ^2^610041 成都，四川大学华西医院肿瘤内科，肿瘤中心; ^2^Department of Medical Oncology, Cancer Center, West China Hospital, Sichuan University, Chengdu 610041, China; ^3^610041 成都，四川大学华西医院肺癌中心/肺癌研究所; ^3^Lung Cancer Center/Lung Cancer Institute, West China Hospital, Sichuan University, Chengdu 610041, China; ^4^610041 成都，四川大学华西医院心理卫生中心; ^4^Mental Health Center, West China Hospital, Sichuan University, Chengdu 610041, China; ^5^610041 成都，四川大学华西医院胸外科; ^5^Department of Thoracic Surgery, West China Hospital, Sichuan University, Chengdu 610041, China

**Keywords:** 肺部多发磨玻璃结节, 自然病程, 全程随访管理, Multiple pulmonary ground-glass nodules, Natural history, Full follow-up management

## Abstract

肺部多发磨玻璃结节（ground-glass nodules, GGNs）的发展变化规律与病程管理是当前肺癌临床研究中的热点与难点。了解肺部多发GGNs自然病程的最新进展，对掌握此疾病的变化规律及制定管理策略有着重要意义，同时利用智能化全程随访管理平台的先进方法，使得GGNs的长期规范化管理成为可能。为此，本文将对肺部多发GGNs自然病程的研究新进展及管理新经验进行综述。

## Abstract (en)

The development and change patterns as well as the disease course management of multiple ground-glass nodules (GGNs) in the lungs are currently hotspots and difficulties in clinical lung cancer research. Understanding the latest advancements in the natural history of multiple GGNs is crucial for grasping the disease variation patterns and formulating management strategies. Meanwhile, utilizing advanced methods such as intelligent follow-up management platforms makes the long-term standardized management of GGNs possible. Therefore, this article provides an overview of the latest research advancements on the natural history of multiple GGNs and new experience in GGNs management.

肺癌是最常见的恶性肿瘤之一，其发病率在我国居所有恶性肿瘤的第1位^[1]^，因此，肺癌筛查及早期诊断至关重要。肺癌筛查指南推荐对高危人群采用低剂量螺旋计算机断层扫描（low-dose computed tomography, LDCT）进行筛查，通过胸部LDCT筛查，无症状肺结节的检出率不断提高，尤其是多发磨玻璃结节（ground-glass nodules, GGNs）的检出增加^[2,3]^。现有资料显示，多发GGNs的患病率占该类结节患者总比例的10%-40%^[4]^，而同时性、持续性多发GGNs的自然病程尚不清楚，高分辨计算机断层扫描（high resolution CT, HRCT）的使用提高了对高危结节的辨别力，对多发GGNs的诊疗提供了有力的帮助^[5]^，但目前仍缺乏精准的治疗手段，利用智能化全程随访管理平台可规律复查，长期随访，为探索多发GGNs的自然病程提供新思路。

## 1 GGNs的基础特征

### 1.1 定义和分类

肺部多发GGNs是指同期出现在肺野内的两个或两个以上最大径≤30 mm的GGNs^[6,7]^。同时性多发GGNs起源于不同部位，无肺外转移，在临床上多发GGNs通常在发现时并无明显相关症状^[8]^。根据存在时间长短进行分类，GGNs可分为一过性和持续性GGNs。一过性而自发消失的短暂GGNs为良性疾病的表现，以炎症性疾病多见；而持续性GGNs在病理类型上多为原位癌或微浸润性腺癌（minimally invasive adenocarcinoma, MIA）^[9,10]^。Son等^[11]^研究了被诊断为原位腺癌（adenocarcinoma in situ, AIS）、MIA或浸润性腺癌的191个切除的GGNs，在病理检查中发现只有38例（20%）为AIS，而61例（32%）为MIA，92例（48%）为浸润性腺癌。

### 1.2 危险因素

肺部同时性、持续性多发GGNs有潜在的恶性可能，需要动态随访观察，目前对于其发病诱因一直处于探索阶段，本文以下探讨的为恶性GGNs的高危因素，良性在此不作讨论。众所周知，肺癌的高危致病因素之一是吸烟，但是吸烟是否对多发GGNs的发展产生影响，说法不一。调查所知大多数有GGNs的患者不吸烟，吸烟可能不是GGNs的致病因素^[12]^，与吸烟诱导的肺癌不同，GGNs的发生与吸烟关系不大。

有研究^[13]^表明，恶性肿瘤家族史可增加结节的恶性风险以及结节增长的风险，但在另一项Kim等^[14]^的研究中显示，恶性肿瘤家族史相关性差异并没有发现有统计学意义，家族史并不是GGNs的重要影响因素，故恶性肿瘤家族史对多发GGNs的影响有待深入研究。Hosgood等^[15]^的研究表明，在国内室内空气污染以及烹饪引起的烟雾是肺部疾病的因素。Bhurosy等^[16]^的研究表明，接触烹饪烟雾最近成为一个潜在的发生肺部肿瘤的危险因素，但并没有确切的证据支持这一说法。由此可以看出，上述研究观点依然不能提供多发GGNs形成的原因，需后期进一步进行相关的探索。

### 1.3 基因特征

有关多发GGNs的基因组数据有限，基因分析对多发GGNs的研究、临床诊断起着重要的作用，有助于结节活检后识别高危患者，基因遗传学的研究显示多发GGNs患者的基因驱动突变存在高度差异性，这些肿瘤大多是多灶性、独立的癌症病灶，而非肺内转移病灶^[17]^。随着基因检测的广泛应用，基因组特征在多原发肺癌中的诊断和分子分型中发挥重要作用^[18,19]^，当病灶间没有共同的驱动基因突变时可判别为多原发肺癌^[20,21]^。在多发GGNs中，51.7%的GGNs会出现表皮生长因子受体（epidermal growth factor receptor, EGFR）突变^[22]^，目前常检测的以EGFR、Kirsten大鼠肉瘤病毒癌基因同源物（Kirsten rat sarcoma viral oncogene homolog, KRAS）、间变性淋巴瘤激酶（anaplastic lymphoma kinase, ALK）重排等常见的基因来区分，如在主要病灶有EGFR突变的病例中，次要病灶中EGFR突变占41.6%；主要病灶未检出EGFR突变的病例中，次要病灶均未检出EGFR突变^[23]^。多发GGNs发病机制不明确，为诊断标准及治疗选择增加了复杂性。随着基因测序技术的不断完善及应用，将有助于解析多发GGNs的机制。

## 2 GGNs的自然病程

肺部多发GGNs的长期自然病程尚不清楚。研究^[24]^显示，多发GGNs属于惰性生长，与实性结节相比其生长速度较为缓慢。在Obayashi等^[25]^研究中依据病理组织学亚型的不同，对371例手术切除的原发性肺癌进行回顾性分析发现，所有具有磨玻璃样成分的原发性肺癌均为腺癌，其体积倍增时间最长，无论体积倍增时间是基于整个肿瘤直径还是基于实变直径来计算，具有磨玻璃成分的腺癌中体积倍增时间明显长于不具有磨玻璃成分的腺癌（中位体积倍增时间：725 vs 177 d），这表明GGNs体积倍增时间与结节的恶变有关。

在Kakinuma等^[26]^的一项对肺部亚实性结节（subsolid nodule, SSN）自然发展过程的前瞻性研究中，对首次CT检查持续3个月的SSN，结节的长轴直径≤3 cm，实性成分长轴直径≤5 mm的结节纳入研究，共795例患者的1229个SSNs开展了评估为浸润性腺癌的频率。SSN分为3类，包括纯磨玻璃结节（pure ground-glass nodule, pGGN）、异质磨玻璃结节（heterogeneous ground glass nodule, hGGN）（仅在肺窗检测到固体成分）和部分实性结节。平均（4.3±2.5）年的随访中，根据SSNs的基线水平区分：1046例pGGN中13例（1.2%）发展为hGGN，56例（5.4%）发展为部分实性结节。在最终的随访中，977例pGGN中，有35例行手术切除，术后病理显示9例MIA、21例AIS和5例非典型腺瘤性增生；78例hGGN中有7例行手术切除，术后病理显示5例MIA和2例AIS；174例部分实性结节中有49例行手术切除，术后病理显示12例浸润性腺癌、26例MIA、10例AIS和1例腺瘤性增生。上述研究结果提示GGNs的发展过程依次是pGGN、肺窗可见实体成分、肺窗及纵隔窗两者可见实体成分，GGNs发展过程呈惰性生长。Kim等^[14]^在对行肺部切除术后患者GGNs的研究中比较了实性成分增加的GGNs与无实性成分的GGNs的特点，结果显示部分实性GGNs与结节生长显著相关，其他因素与结节增大无明显关系，故实性成分的出现是预测自然生长的因素。Zhang等^[27]^和范子文等^[28]^的研究结果显示，肺部GGNs的生长受到实性成分比例的影响，此外，在GGNs的大多数研究^[27,29,30]^中，初诊时GGNs的直径是临床预测结节生长的指标之一。

在Sato等^[24]^的研究中共纳入187例患者，其中78例患者为多发GGNs，25例患者在36个月时发生GGNs进展，研究结果显示多发GGNs患者中结节≥10 mm以及既往肺癌病史是36个月进展的危险因素，36个月是最佳观察期，其中肺癌病史和初始GGNs结节>10 mm是多发GGNs结节生长的危险因素。上述研究中可以对多发GGNs的自然病程有清晰的认识，GGNs的生长属于惰性生长，受自然病程受体积倍增时间、实性成分占比、初诊时GGNs直径和肺癌病史等多种因素的影响。明确持续性存在的多发GGNs自然病程对临床医生诊疗策略及全程随访管理的制定至关重要。

## 3 GGNs的诊疗策略

随着肺癌筛查的普及，多发肺结节的检出率越来越高，对高危结节的辨识也越来越精准。有文献^[6,31]^报道多发GGNs占筛查GGNs的22%-48.5%。Farjah等^[32]^对成人偶然发现肺结节的回顾性研究显示多发GGNs占比57%。多发GGNs可能位于同侧多个肺叶或异侧多个肺叶，每个肺叶都有结节，且每个结节都可能具有不同的病理类型，无疑给临床诊治带来更大的挑战，目前对于多发GGNs的最佳治疗方案仍需要长时间的临床研究。

### 3.1 手术诊疗策略

Fleischner学会在2017年的指南^[33]^中指出，对于多发GGNs结节直径<6 mm在3-6个月内复查CT无明显变化，需2-4年再次复查CT；多发GGNs结节直径>6 mm在3-6个月内复查CT，之后主要是对有高度恶性的结节进行长期随访。但目前多发GGNs的处理原则在于优先处理危险度最高的结节^[34]^。根据结节的大小、位置和数量，对于肺功能良好、既往体健、无基础性疾病的患者，手术治疗是目前首选方式。相对于单发GGN，多发GGNs进行手术时需要更加周密的手术方式考量^[35,36]^。Zhang等^[37]^研究认为，对于疑似的亚实性结节、同侧易触及的GGNs或随访期间对侧GGNs增大或者实性成分增加的患者，应该考虑手术治疗。多发GGNs切除术后预后较好，5年生存率可达90%以上，即使是局部切除术也不影响预后^[38]^。在多发GGNs的治疗原则中，对新发的GGNs单病灶，建议亚肺叶切除手术治疗为主，而Suzuki等^[39]^研究提出“打鼹鼠”策略治疗多发GGNs，即在随访过程中某个结节出现变化有恶性倾向时，需处理该结节，其他结节继续随访。针对多发GGNs在切除主要病灶后肺内残留结节的处理仍然存在争议，目前大部分遵从切除主要病灶后，选择性切除或者全程随访管理其余结节的变化^[40]^。

### 3.2 非手术诊疗策略

肺功能差、有呼吸系统疾病、年龄大、严重出血倾向、基础疾病多，不能行手术治疗的患者可考虑肿瘤热消融治疗^[41,42]^。对于散在多个肺叶的磨玻璃影为主的多发病灶，首选手术切除主要病灶和有限切除易触及的实性成分多的结节，对于直径不大于10 mm或未出现实体部分的病灶，采用立体定向放射治疗（stereotactic body radiotherapy, SBRT）或术后全程监测^[43]^。病灶多表现为GGN的同时性多原发肺癌患者，肺内未切除的GGN发生进展时，持续性的全程随访联合手术治疗或者SBRT是目前常用的治疗策略。对于术后新发多病灶诊疗，应全面评估心肺功能情况，建议多学科会诊（multi-disciplinary treatment, MDT）和医患共同决策患者治疗方案^[44,45]^，但在临床实践中患者往往更倾向于保守治疗，而不是手术或者放疗。有个案报道^[46]^EGFR突变型多原发性肺癌患者在首次手术后残留多个GGNs病灶，进行了EGFR酪氨酸激酶抑制剂（tyrosine kinase inhibitor, TKI）治疗，预后良好，这对于不适合或者不愿意再接受一次手术的患者可能是一种有前景的治疗方法。

多发GGNs的诊疗策略还未出现统一随访标准，目前国内针对于肺部多发GGNs的临床指南建议在治疗方面术前采用MDT会诊模式及医患共同决策来制定个体化治疗方案^[42]^。

## 4 GGNs的全程随访管理策略研究进展

由于胸部CT广泛应用于肺癌早期的筛查和健康体检，多发GGNs的检出率明显提高，但目前仍缺乏精准的治疗手段。目前大部分的肺结节全程管理模式都是针对单发肺结节的随访，而多发GGNs目前还处于探索阶段，没有专门的管理模式，多发GGNs的临床管理在诊治中有着非常重要的作用。

### 4.1 多发GGNs的全程管理模式的理念

研究多发GGNs的自然病程有助于对肺结节良恶性进行判断，但GGNs属于惰性生长，目前研究^[47]^表明GGNs的自然病程受体积倍增时间、实性成分占比、初诊时GGNs直径和肺癌病史等多种因素的影响。Sato等^[24]^的研究分析了多发GGNs和单发GGN的自然病程，在随访36个月时多发GGNs和单发GGN的进展时间无明显差异，这表明多发GGNs可以借鉴单发GGN的随访管理策略。在进行随访管理中需要考虑每个结节的恶性程度，根据GGN的恶性程度制定更具体、更符合诊治的临床管理策略。

### 4.2 多发GGNs的全程管理模式的实践

基于实现肺结节高效筛查、科学诊疗的全程随访模式^[48]^，四川大学华西医院创建了肺结节/肺癌患者全程管理平台，与此同时在平台的设计与应用中，四川大学华西医院深化“互联网+”的医护管一体化肺结节/肺癌全程管理，通过组建多学科临床和管理团队，利用互联网以及人工智能工具实现风险分级，对不同风险层级肺结节患者进行主动分流，执行分层管理方案，对低危肺结节患者进行健康教育推送及基础随访管理；对中危肺结节患者进行线上线下门诊诊疗相结合，通过智慧化管理系统平台、智能语音、智能短信等多途径提醒患者遵医嘱，规律随访，建立个体化诊疗随访方案；对高危肺结节患者主动匹配临床医生和诊疗协助，或发起多学科协同诊疗^[49]^，该文中提出的随访管理模式为管理多发GGNs患者提出了新方向。众所周知，多发GGNs无法一次性手术切除，肺部剩余的结节定期随访尤为重要，同时可以避免对GGNs的过度诊断和过度治疗^[50]^。目前互联网和人工智能工具实现肺结节智能风险升级随访管理，越来越多的患者在CT的升级下，多发GGNs检出率不断升高，人群基数大，智能化随访信息系统的运用及专病管理师岗位的设置为多发GGNs提供了新的管理模式，减少随访脱落次数及失访率，提高了患者对医院的总体满意度^[51]^。王乔利等^[52]^的多学科肺结节管理模式提出，通过主动地、长期地随访肺结节患者，多学科协助运行，防止遗漏或者延误，及时追踪患者的病情变化，能更有效地提高肺结节的诊治效果。

上述针对结节的随访管理模式的设计与应用，为探索多发GGNs管理模式提供了新的思路，尤其对自然病程的全程随访管理更是少见，但自然病程在对了解多发GGNs的发病机制及临床诊治方面发挥着重要的作用，利用智能化全程随访管理平台，长期规律随访每个结节的变化，参与患者管理及规律复查，为实现最佳临床诊治打下基础。

### 4.3 多发GGNs全程管理的困难与挑战

对于多发GGNs的随访管理策略除了关注主要病灶及结节形态外，还应考虑性别、年龄、家族史、既往病史等危险因素。自然病程目前尚未有清晰的认识，如何根据自然病程选择精细的管理模式、寻找最佳治疗时机、防止过度诊治还有待研究。

## 5 总结

随着计算机技术的普及，肺部同时性、持续性多发GGNs检出率增高，目前治疗方法有限。在临床手术同时切除全部结节行不通，多发GGNs较之于单发GGN存在着差异，在处理的过程中需要采用不同的治疗方式。当今对于多发GGNs的自然病程尚不清楚，很难制定精准的治疗策略。探究引起持续性多发GGNs的危险因素，降低多发GGNs的发病率，增加患者对多发GGNs的认识，减少多发GGNs给患者带来的身体和心理负担，对患者进行长期的随访管理是非常必要的。

目前，对于多发GGNs的认识有限，围绕多发GGNs的知识存在巨大缺口，随着个体化、精准化医疗的不断发展，完善多发GGNs的个体化、精准化诊疗，需要通过多中心、大样本的前瞻性研究探索其自然病程的发展规律，提出更专业的全程管理策略。
